# Models of Care in Multiple Sclerosis: A Survey of Canadian Health Providers

**DOI:** 10.3389/fneur.2022.904757

**Published:** 2022-05-20

**Authors:** Ruth Ann Marrie, Sarah J. Donkers, Draga Jichici, Olinka Hrebicek, Luanne Metz, Sarah A. Morrow, Jiwon Oh, Julie Pétrin, Penelope Smyth, Virginia Devonshire

**Affiliations:** ^1^Department of Internal Medicine, Max Rady College of Medicine, Rady Faculty of Health Sciences, University of Manitoba, Winnipeg, MA, Canada; ^2^Department of Community Health Sciences, Max Rady College of Medicine, Rady Faculty of Health Sciences, University of Manitoba, Winnipeg, MA, Canada; ^3^School of Rehabilitation Science, College of Medicine, University of Saskatchewan, Saskatoon, SK, Canada; ^4^Department of Critical Care Medicine and Neurology, Hamilton Health Sciences, McMaster University, Hamilton, ON, Canada; ^5^Royal Jubilee Hospital, Victoria, BC, Canada; ^6^Department of Clinical Neurosciences, Cumming School of Medicine, University of Calgary, Calgary, AB, Canada; ^7^Department of Clinical Neurological Sciences, Western University, London, ON, Canada; ^8^Division of Neurology, St. Michael's Hospital, University of Toronto, Toronto, ON, Canada; ^9^Department of Medicine and Neuroscience and Mental Health Institute, University of Alberta, Edmonton, AB, Canada; ^10^Department of Medicine (Neurology), University of British Columbia, Vancouver, BC, Canada

**Keywords:** multiple sclerosis, models of care, multidisciplinary, Canada, survey

## Abstract

**Objective:**

Little work has evaluated integrated models of care in multiple sclerosis (MS) and the composition of MS care teams across Canada is largely unknown. We aimed to gather information regarding existing models of MS care across Canada, and to assess the perceptions of health care providers (HCPs) regarding the models of care required to fully meet the needs of the person with MS.

**Methods:**

We conducted an anonymous online survey targeting Canadian HCPs working in MS Clinics, and neurologists delivering MS care whether or not they were based in an MS Clinic. We queried the types of HCPs delivering care within formal MS Clinics, wait times for HCPs, the perceived importance of different types of HCPs for good quality care, assessments conducted, and whether clinic databases were used. We summarized survey responses using descriptive statistics.

**Results:**

Of the 716 HCPs to whom the survey was distributed, 100 (13.9%) people responded. Of the 100 respondents, 85 (85%) indicated that their clinical practice included people with MS and responded to specific questions about clinical care. The most common types of providers within MS Clinics with integrated models of care were neurologists and MS nurses. Of 23 responding MS Clinics, 10 (43.5%) indicated that there were not enough neurologists, and 16 (69.6%) indicated that there were not enough non-neurologist HCPs to provide adequate care. More than 50% of clinics reported wait times exceeding 3 months for physiatrists, physiotherapists, psychiatrists, psychologists, neuropsychologists and urologists; in some clinics wait times for these providers exceeded 1 year. Multiple disciplines were identified as important or very important for delivering good quality MS care. Over 90% of respondents thought it was important for neurologists, nurse practitioners, MS nurses and psychiatrists to be co-located within MS Clinics.

**Conclusion:**

Canadian HCPs viewed the ideal MS service as being multidisciplinary in nature and ideally integrated. Efforts are needed to improve timely access to specialized MS care in Canada, and to evaluate how outcomes are influenced by access to care.

## Introduction

Multiple sclerosis (MS) is a chronic, immuno-inflammatory disease of the central nervous system affecting over 90,000 Canadians, and more than 2.8 million persons worldwide (1, 2). MS is a complex chronic disease characterized by relapses and progression of physical and cognitive impairment over time. Comorbid conditions such as depression and anxiety disorders are also common. MS has a negative effect on employment status (3), health-related quality of life (4–7), and the ability to perform personal and instrumental activities of daily living (ADL).

Comprehensive management of MS typically involves treatment of acute relapses, disease-modifying therapy (DMT) to modify the course of the disease by reducing relapses and disability progression, chronic symptom management, supports in regards to coping and function, and education. The National Collaborating Centre for Chronic Conditions at the Royal College of Physicians (United Kingdom, UK) developed a national clinical guideline for diagnosis and management of multiple sclerosis in primary and secondary care (8). These guidelines included a recommendation that people with MS have access to specialist rehabilitation services to assess complex problems which cannot be evaluated by a single team member and to provide an integrated program of rehabilitation, to monitor change, and to advise other members of the health care team. Integral components of the team were a physician, nurse, physiotherapist, occupational therapist, social worker, speech and language therapist, and clinical psychologists, consistent with recommendations for an MS Care Unit proposed by Sorensen et al. (9). A recent review suggested that multidisciplinary rehabilitation improves activity, participation and quality of life (10).

Multiple models of care exist for the management of chronic diseases such as MS. These include shared care models, primary-care specialist referral models, and specialized multi-disciplinary team-based models. Integrated models of care are those in which multiple health care providers are co-located and collaboratively manage patients, but relatively little work has evaluated integrated models of care in MS (11). In Canada, government-funded, specialized MS Clinics exist in most provinces, in part because government-funded access to MS-specific DMTs often requires assessment by a neurologist with specific expertise in MS. The composition of MS care teams across Canada is largely unknown, including whether the teams involve an integrated care model, and what disciplines are involved. Access to those teams, as assessed using the wait times are for each discipline, are also unknown. This information is important to inform policy development and resource allocations aimed at improving access to care and disease outcomes.

We aimed to gather information regarding existing models of MS care across Canada, and to assess the perceptions of health care providers regarding the models of care required to fully meet the needs of the person with MS. We hypothesized that models of MS care would vary with respect to their components (that is, what health care disciplines are considered to be part of the MS team), and structure (that is, whether team members and services are fully integrated and co-localized, integrated but not co-localized, not integrated or co-localized). We further hypothesized that MS health care providers (HCP) would identify a broad range of disciplines as being needed to support high quality care for persons with MS.

## Methods

We report the design and findings of this study according to the Consensus-Based Checklist for Reporting of Survey Studies (CROSS) (12).

### Setting

This study was conducted in Canada, a country with a population of >38 million, distributed over 10 provinces and 3 territories. Health care in Canada is universal, and publicly funded for essential services, including hospitalizations and physician visits. Because health care is organized and delivered at the provincial level, variation exists in the services available and in how they are delivered. Thus, care from non-physician providers such as psychologists and physical therapists is often not covered except through specific disease-oriented programs, such as MS Clinics. Private health insurance plans may be used to obtain coverage for services not paid for by the universal health system.

### Design and Population

This was a cross-sectional study utilizing an anonymous online survey. We targeted two populations, both comprised of HCPs practicing in Canada who were currently delivering MS care. The first population was neurologists, whether or not they practiced in the setting of an MS Clinic, given their critical role in diagnosis of MS and their role in access to DMTs. The second population was providers of all disciplines working within MS Clinics. To create the survey distribution list for neurologists, we collated names of health care professionals in Canada from multiple sources including Medical Directors of provincial MS Clinics, the Canadian Network of MS Clinics (a national network of academic and community-based clinics for MS care), provincial college of physician listings, and the American Academy of Neurology member directory. We used the provincial college listings, and the American Academy of Neurology member directory to enhance identification of neurologists practicing in Canada who might deliver MS care outside formally labeled MS Clinics. Medical Directors of MS Clinics assisted with identification of non-neurologist HCPs working in MS Clinics. The University of Manitoba Health Research Ethics Board and Shared Health approved the study. The survey included a consent statement indicating that completion of the survey implied consent.

### Survey

We adapted an existing questionnaire that assessed models of care in inflammatory bowel disease (IBD), another chronic immune-mediated disease that often requires multidisciplinary care (13). The survey assessed characteristics of the respondent, their work settings, types of HCPs delivering care within formal MS Clinics, the perceived importance of different types of HCPs for good quality care, clinic databases and assessments conducted. Supplementary Appendix I includes the full questionnaire. The questionnaire was pilot tested by two individuals who were not involved in survey development prior to distribution.

#### Respondent Characteristics

Respondent characteristics queried included age, gender, discipline, whether they had a particular interest in MS, if they had fellowship training in MS and whether their clinical practice included people living with MS. Disciplines included neurologist, physiatrist, MS Nurse, nurse practitioner, physician assistant, physiotherapist, occupational therapist, social worker, psychologist, psychiatrist/neuropsychiatrist, neuroradiologist/radiologist, dietician, urologist/urogynecologist, general ophthalmologist, neuro-ophthalmologist, speech-language pathologist, pharmacist, neuropsychologist, and other. We did not include primary care providers as they are not integrated within MS Clinics in Canada. Respondents who indicated that their clinical practice did not include people living with MS were not asked any further questions, and were excluded from the primary analysis.

#### Work Setting

The remaining respondents were asked to provide details regarding their MS-related work including their training, length of time working in the MS field, the setting of their MS practice, what percentage of their clinical work concerns MS, practice size, whether they treated adults or children with MS, whether they worked within a formally labeled MS Clinic (and if so, which one); and whether they considered their MS service to apply an integrated model of care.

#### Composition of MS Clinics and Timeliness of Care

To limit response burden, questions regarding services available within formally labeled MS Clinics were answered by a single respondent who had been designated to do so in advance of that survey through contact with the Medical Director of the clinic. We asked whether the MS Clinic used an integrated model of care (model in which several HCPs are located at the same site and manage patients collaboratively), and which types of HCPs worked in the clinic. For each provider indicated as working in the clinic, respondents indicated the wait time for a new referral (0–3, 4–6, 7–12, >12 months), as well as the total number of HCPs and total full-time equivalents (FTE) for each type of HCP? For all MS services, whether or not they were formally labeled or integrated, we asked respondents to indicate which publicly funded types of HCPs were accessible outside their MS service, as well as the wait times for a new referral. Respondents also indicated whether the number of neurologists (FTEs), and non-neurologist HCPs at the MS Clinic allowed for provision of optimal care.

#### Clinical Assessments and Referral Patterns

Given the high prevalence of mood and anxiety disorders among people with MS, we asked if providers routinely asked about stress, anxiety, or depression during their encounters with patients (yes/no). If yes, they were asked if this was by verbally asking questions, using a questionnaire or other means. We also asked about the use of standardized assessments not related to mental health, focusing on those which are widely recognized, accessible and validated for use in MS, including the Timed 25 Foot Walk, Nine Hole Peg Test, a measure of processing speed including the Symbol Digit Modalities Test, the Expanded Disability Status Scale (EDSS) score, a measure of quality of life (specify), and screening measures for depression and anxiety disorders including the Patient Health Questionnaire-9, Hospital Anxiety and Depression Scale, Clinical Epidemiology Studies Depression scale, Beck Depression Inventory, Generalized Anxiety Disorder-7, OASIS, PROMIS Depression and PROMIS Anxiety measures. An “other” option was provided for respondents to specify other assessment measures used.

Respondents reported the approximate percentage of their MS patients they referred to the following health professionals: physiotherapist, occupational therapist, social worker, psychologist, psychiatrist, neuropsychologist, and dietitian. These providers were selected based on the high prevalence of comorbid mental health disorders in people with MS (14), the benefits of multi-disciplinary rehabilitation in MS (10), and recommendations for MS care in the UK (8).

#### Database Information

Quality improvement requires the ability to measure processes and outcomes. Therefore, we asked the designated responder within MS Clinics “Does your clinic currently collect the following data electronically (clinic database or administrative data) to allow determination of outcomes?”, including date of symptom onset, date of first neurologist encounter, date of each MRI after symptom onset, date of first MS Clinic visit, date of first DMT discussion, date of first DMT initiation, date of diagnosis, date DMT insurance effective, dates of each visit, date of each EDSS, dates of each care provider encounter and who provided care, dates of each DMT started and stopped, reason for DMT switch, dates and scores of each cognitive test (and which test), dates of each relapse, referral to MS Clinic date, reason for referral, whether the referral was internal to the institution or external, and the health professional who referred. For each item we asked if the information was collected at the clinical level or for each physician. If an item was collected, we asked the completeness and accuracy of the information using visual analog scales marked low/medium/high.

#### Perceived Ideal Models of Care

Respondents indicated “How important are these types of health professionals for good quality MS care?” on Likert-type response scale (not at all important, unimportant, neither important nor unimportant, important, very important). If a health professional was identified as important or very important a follow-up question asked how important it was to good quality care that they work within the MS Clinic using the same Likert-type response scale. The survey closed with two open-ended questions asked respondents to (i) Describe the ideal MS service; and (ii) What resources would be most helpful in improving MS care at your clinic.

### Survey Administration

The survey was developed and managed using REDCap electronic data capture tools hosted at the University of Manitoba. REDCap (Research Electronic Data Capture) is a secure, web-based software platform designed to support data capture for research studies (15). The survey was distributed beginning in mid-September 2021 and closed January 31, 2022. Prior to questionnaire distribution, members of the Canadian Network of MS Clinics were advised via email that the survey was going to be distributed. Initially, the individual survey links were distributed directly using the REDCap survey distribution tools. However, it became apparent that email invitations issued via the REDCap server were sometimes being treated as junk/spam emails. To address this problem reminders were manually generated and sent from the institutional email address of a study coordinator at least three times. Two general reminders were also issued through the Canadian Network of MS Clinics listserv.

### Analysis

We summarized the responses to survey questions using descriptive statistics including mean [standard deviation (SD)], median [interquartile range (IQR)], and frequency (percent). Missing data were not imputed. Bivariate analyses tested the association between respondent characteristics and models of care using chi-square tests, Fisher's exact tests, and non-parametric measures of association as appropriate. Formal qualitative analysis of the responses to the open-ended questions will be reported separately.

The analysis was conducted using SAS V9.4.2 (SAS Institute Inc., Cary, NC).

## Results

### Participant Characteristics

Overall, of 716 to whom the survey was distributed, 100 (13.9%) people responded. Of the 100 respondents, 85 (85%) indicated that their clinical practice included people with MS and were presented with specific questions about MS care (Table 1). The demographic characteristics of respondents were similar for those whose practices did and did not include people with MS. Respondents whose practice included people with MS constitute the study sample used for the remaining analyses.

**Table 1:** Characteristics of respondents, stratified according to whether practice includes people with multiple sclerosis.

	**Practice includes MS**		
**Characteristic**	**No**	**Yes**	*P*-value^*^
	**(*N* = 13)**	**(*N* = 85)**	
Age (yrs), mean (SD)	51.4 (13.8)	47.6 (11.4)	0.28
Gender, *n* (%)			
Male	6 (46.2)	34 (40.0)	0.86
Female	7 (53.8)	50 (58.8)	
Prefer not to answer	0 (0)	1 (1.2)	
Discipline, *n* (%)			
Neurologist	12 (92.31)	57 (67.06)	0.76
Physiatrist	0 (0)	2 (2.35)	
MS nurse	0 (0)	7 (8.24)	
Nurse practitioner	0 (0)	4 (4.71)	
Physician assistant	0 (0)	1 (1.18)	
Physiotherapist	0 (0)	3 (3.53)	
Occupational therapist	0 (0)	3 (3.53)	
Social worker	0 (0)	3 (3.53)	
Psychologist	0 (0)	1 (1.18)	
Neuropsychiatrist	0 (0)	2 (2.35)	
Neuropsychologist	0 (0)	1(1.18)	
Other (MS educator, administrator)	1 (7.69)	1 (1.18)	
Particular interest in MS, *n* (%)	3 (23.08)	78 (91.76)	**<0.001**
Fellowship Training in MS, *n* (%)	-	39 (50.0)	0.089
No. years following training involved in MS Care, median (p25–p75)		13 (5–20)	
Province, *n* (%)			
British Columbia	–	12 (14.29)	
Alberta		14 (16.67)	
Saskatchewan		4 (4.76)	
Manitoba		21 (25.0)	
Ontario		22 (26.19)	
Quebec		6 (7.14)	
New Brunswick		2 (2.38)	
Nova Scotia		3 (3.57)	
Work setting^b^, *n* (%)			
General hospital		19 (22.9)	
University hospital		57 (68.7)	
Solo private practice		4 (4.8)	
Group private practice		3 (3.6)	
Work in formally labeled MS Clinic^a^, *n* (%)		63 (5.0)	
Age of MS population treated, *n* (%)			
Adults	–	5 (89.29)	
Children (≤16 years)	–	2 (32.14)	
Percentage of clinical work that concerns MS, median (p25–p5)		0 (30–90)	
No. MS patients per week, median (p25–p5)		20 (6–30)	
No. MS patients in practice, median (p25–p5)		400 (50–950)	

a *missing*;

b *indicated other but did not specify. Bold indicates statistical significance*.

* *Comparing No vs. Yes*.

Among the 85 respondents, most were neurologists (*n* = 57), followed by MS nurses (*n* = 7); slightly over half were female. All other types of HCPs had ≤3 respondents. Eight out of the ten Canadian provinces were represented, and we received responses from 26 (76.5%) of the 34 formally labeled MS Clinics.

### Work Setting

Overall, the median (IQR) practice size was 400 (50–950) people with MS, but this varied by discipline. Among neurologists, median (IQR) practice size was 425 (75–800), whereas it was much larger for MS nurses [1,600 (500–4,000), *p* = 0.016] and similar for nurse practitioners [300 (40–500), *p* = 0.44]. The number of respondents for other disciplines limited inference about practice size.

### Composition of MS Clinics and Timeliness of Care

Although 63 respondents who reported working in an MS clinic, only 26 (1 per site) were designated respondents for this group of questions. Of these 26, 21 (80.8%) respondents reported that they worked within an integrated model of care, and 1 respondent did not indicate the model of care. The most common types of providers within MS Clinics with integrated models of care were neurologists and MS nurses, with 20 of 21 MS Clinics reporting that they had both types of HCPs. One clinic reported having neither neurologists nor MS nurses. After neurologists and MS nurses, physiatrists, psychologists, and physiotherapists and occupational therapists were the most common HCPs (Table 2).

**Table 2:** Availability of health care providers to multiple sclerosis (MS) Clinics.

**Type of provider**	Non-integrated (*n* = 4^*^)	**Integrated (*****n*** **=** 21)^**^			
		**Outside MS Clinic**	**Within MS Clinic**	**# within MS Clinic**	**# FTEs within MS Clinic**
Neurologist	4 (100)	12 (60.0)	20 (95.2)	4 (3.5–5.5)	2.5 (2.0–3.0)
MS nurse	4 (100)	3 (15.0)	20 (95.2)	3 (1–4)	2.0 (1.2–2.5)
Nurse practitioner	1 (25.0)	5 (25.0)	10 (47.6)	1 (1–1)	1 (1–1)
Physician assistant	0 (0)	1 (5.3)	4 (19.1)	4 (1–7)	1 (1–1)
Physiotherapist	2 (50.0)	13 (68.4)	14 (66.7)	1 (1–2)	1 (0.5–1.1)
Occupational therapist^*^	2 (50.0)	11 (57.9)	12 (60.0)	1 (1–2)	1 (0.5–2.2)
Social worker^*^	2 (50.0)	7 (36.4)	5 (25.0)	1 (1–2)	1 (0.5–2.2)
Psychologist	1 (25.0)	9 (47.4)	5 (25.0)	2 (1–4)	0.80 (0.45–1.5)
Psychiatrist^*^	2 (50.0)	10 (52.6)	14 (70.0)	1 (1–2)	0.2 (0.1–1.0)
Radiologist^*^	2 (50.0)	8 (40.0)	13 (61.9)	3 (4–6)	2.5 (0.8–5.0)
Dietitian	2 (50.0)	9 (47.4)	6 (38.6)	1 (1–2)	0.5 (0.3–2.0)
Urologist	2 (50.0)	12 (63.2)	8 (38.1)	2 (1–3)	2.0 (0.2–3.0)
General ophthalmologist	2 (50.0)	16 (84.2)	2 (9.5)	2.5 (2–3)	1.1 (0.2–2.0)
Neuro-ophthalmologist	2 (50.0)	10 (52.6)	11 (52.4)	2 (2–3)	1.5 (0.4–3.0)
Speech language pathologist	2 (50.0)	13 (68.4)	6 (28.6)	1 (1–2)	0.2 (0.2–0.5)
Physiatrist	2 (50.0)	11 (57.9)	15 (71.4)	2 (1–2)	0.4 (0.2–1.9)
Pharmacist	2 (50.0)	10 (55.6)	6 (28.6)	1 (1–1)	1 (1–1)
Neuropsychologist	2 (50.0)	8 (42.1)	9 (42.9)	1 (1–2)	0.75 (0.15–1)
Orthotist	2 (50.0)	13 (68.4)	3 (14.3)	2.5 (2–3)	2 (1–3)

* *n = 1 missing*;

** *Within MS Clinic indicates provider is located within the integrated MS Clinic. Outside MS Clinic indicates provider is not integrated within the MS Clinic but available by referral*.

The most common types of providers available outside those clinics by referral were general ophthalmologists, physiotherapists, orthotists, and speech language pathologists. In MS Clinics without integrated models of care, the most common types of providers that comprised those clinics were neurologists and MS nurses (100%). Availability of all other HCPs (outside those clinics) except nurse practitioners was 50%. Of the 19/21 MS Clinics with integrated models of care who responded to this question, 13/19 (68.4%) reported that they hold multi-disciplinary team meetings, whereas only 1/4 (25.0%) of the non-integrated clinics did so.

**Figure 1:**
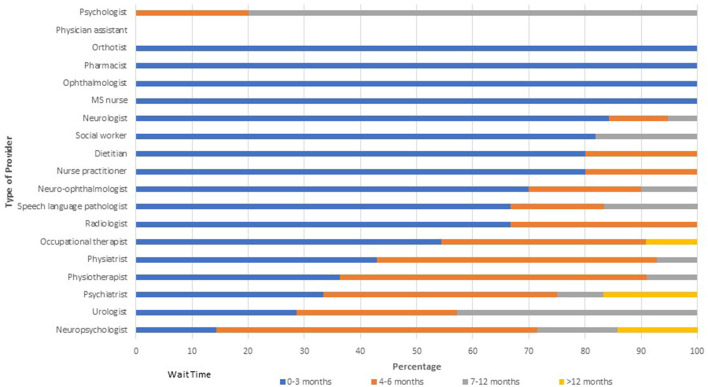
Wait times for providers within multiple sclerosis clinics.

**Figure 2:**
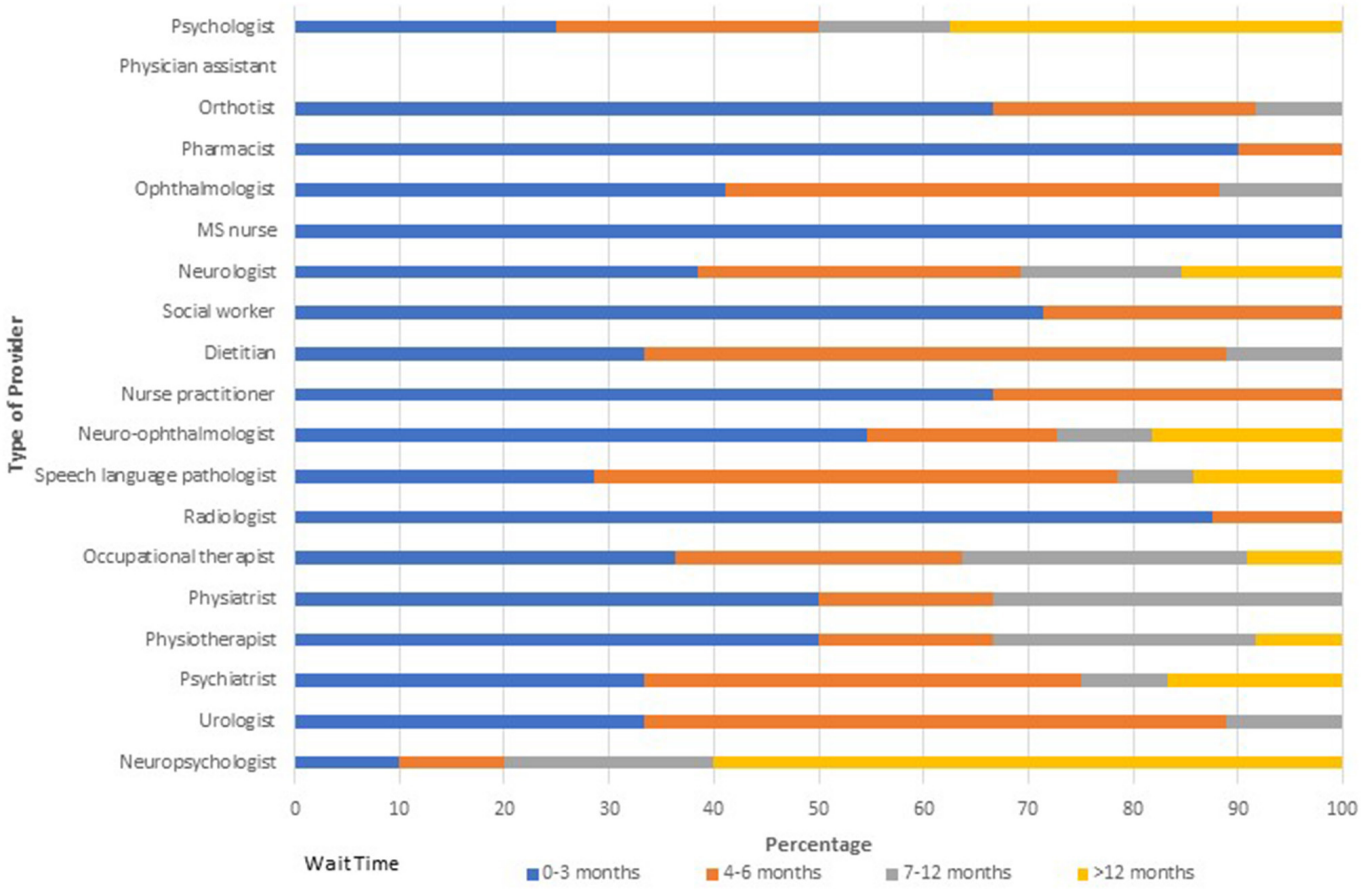
Wait times for providers external to multiple sclerosis clinics.

Of 23 responses, 10 (43.5%) indicated that there were not enough neurologists to provide adequate care, and 16 (69.6%) indicated that there were not enough non-neurologist HCPs to provide adequate care. Wait times for providers within MS Clinics were variable (Figure 1). More than 50% of clinics reported wait times exceeding 3 months for physiatrists, physiotherapists, psychiatrists, psychologists, neuropsychologists and urologists; in some clinics wait times for these providers exceeded 1 year. The only providers who were uniformly accessible within 3 months of referral were orthotists, pharmacists, general ophthalmologists, and MS nurses. Generally, wait times were longer for providers located outside MS Clinics (Figure 2).

### Research and Database-Related Questions

With respect to research, all but one MS Clinic (with an integrated model of care) reported participating in research including 15 (57.69%) clinics reported that they participate in research lead by their team members, and 16 (61.54%) that they participated in research lead by others. Of the 23/26 clinics who responded, 19 (82.6%) indicated that they had a database. The information captured varied across clinics (Supplementary Appendix II). The most commonly captured information was the date of the first clinic visit, dates of other clinic visits, and dates related to initiation and switching of DMT. The least commonly captured information was whether referrals to the MS Clinic were internal or external to the institution and dates DMT coverage became effective. Reported completeness and data accuracy for most data elements captured exceeded 80%, but was lower for dates of DMT coverage, dates of each relapse, and dates of each EDSS.

### Clinical Assessments and Referral Patterns

**Figure 3:**
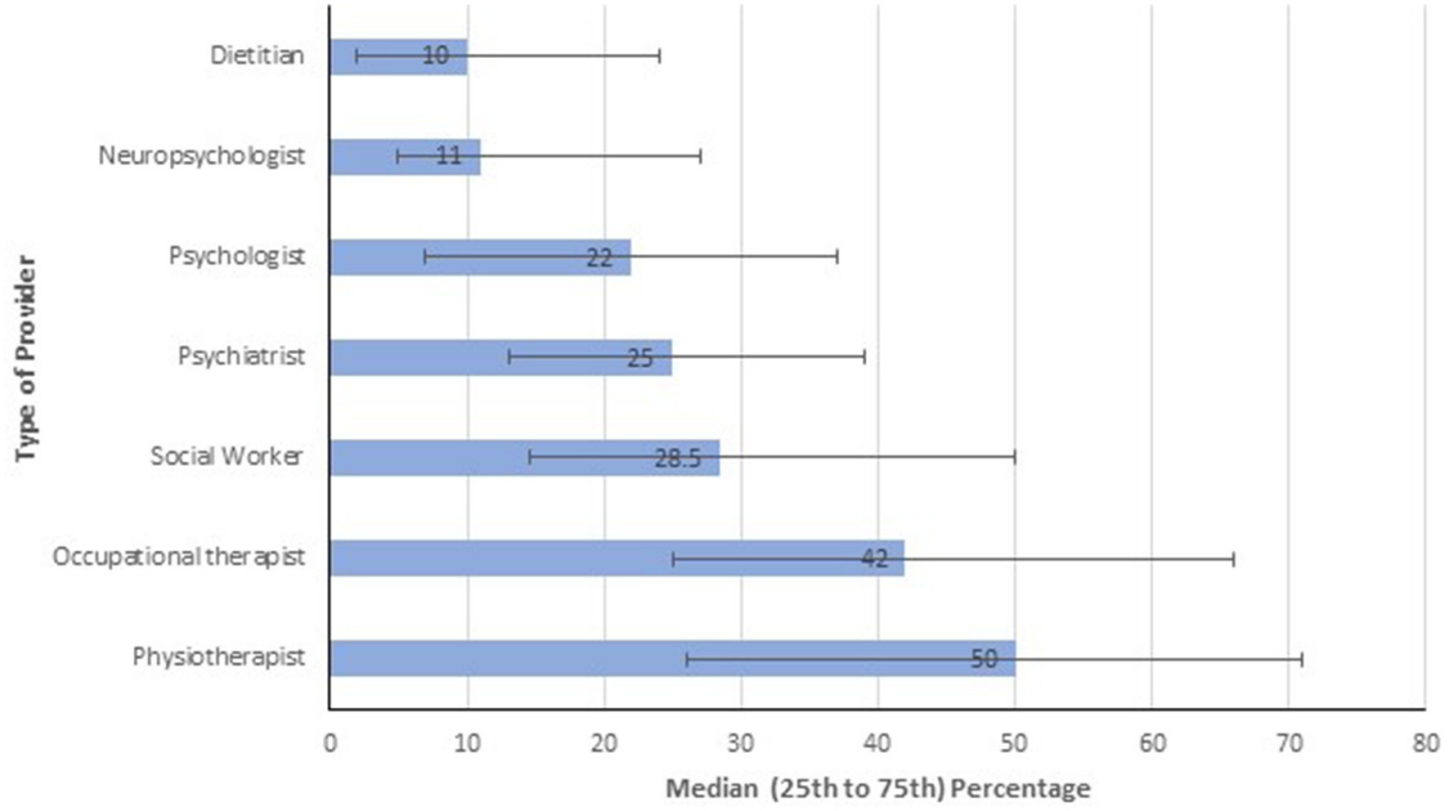
Percentage of patients seen referred to different types of providers.

All respondents provided information regarding referral patterns, clinical assessments and ideal models of care. Overall, HCPs most often referred to physiotherapists, followed by occupational therapists (Figure 3). Although the findings should be interpreted with caution due to small numbers for HCPs other than neurologists, physiatrists (*n* = 2) were more likely to refer to physiotherapy (85 vs. 50%, *p* = 0.11) and occupational therapists (71.5 vs. 40.5%, *p* = 0.035) than neurologists. Occupational therapists (*n* = 3) were similarly more likely to refer to physiotherapists (71%, *p* = 0.051) as well as social workers (67%, *p* = 0.043) than neurologists.

The most common routinely performed assessment was the EDSS whether care was provided within or external to a formally labeled MS Clinic (Table 3), followed by the timed 25-foot walk and the nine hole peg test. Assessment with a timed 25-foot walk or nine-hole peg test was statistically significantly more common within an MS Clinic. When we restricted the analysis to neurologists, the differences with respect to the timed 25-foot walk or nine-hole peg test were larger, and screening of cognition with a processing speed test of some kind was more common within MS Clinics. Two providers (neurologist, occupational therapist) reported that they used the Montreal Cognitive Assessment when applicable. Other assessments reported included the BERG Balance Scale (physiotherapist, *n* = 1), grip strength (physiotherapist, *n* = 1), Modified Fatigue Impact Scale (occupational therapist, *n* = 1), Adolescent Adult Sensory Profile (occupational therapist, *n* = 1), measures of visual function (neurologist, *n* = 1), the Godin Leisure Time Activity Questionnaire (neurologist, *n* = 1), and a locally developed questionnaire (neurologist, *n* = 1). Assessment of quality of life was uncommon. Instruments used to assess quality of life included the Health Utilities Index Mark-3 (*n* = 2), the PEDS-QoL (*n* = 2), and MS-specific instruments (*n* = 2).

**Table 3:** Percentage of assessments routinely performed stratified by whether the health care provider is in an integrated multiple sclerosis clinic or not.

**Assessment**	**Integrated clinic^a^**		***P*-value**
	**No (*n* = 21)**	**Yes (*n* = 63)**	
*All providers including neurologists*			
Nine hole peg test	1 (4.8)	23 (36.5)	0.0048
Timed 25-foot walk	6 (28.6)	40 (63.5)	0.0056
SDMT or PST	6 (28.6)	30 (47.6)	0.13
EDSS	1 (4.8)	9 (14.3)	0.44
HRQOL	2 (9.5)	6 (9.5)	1
Depression questionnaire	5 (23.8)	13 (20.6)	0.76
Anxiety questionnaire	2 (9.5)	7 (11.1)	1
*Neurologists*	*n* = 16	*n* = 40	
Nine hole peg test	0 (0)	12 (30.0)	0.012
Timed 25-foot walk	4 (25.0)	26 (65.0)	0.0087
SDMT or PST	3 (18.8)	18 (45.0)	0.078
EDSS	11 (68.8)	5 (90.0)	0.1
HRQOL	1 (6.3)	4 (10.0)	1
Depression questionnaire	3 (18.8)	8 (20.0)	1
Anxiety questionnaire	1 (6.3)	4 (10.0)	1

*SDMT, Symbol Digit Modalities Test; PST, processing speed test; EDSS, Expanded Disability Status Scale score; HRQOL, health-related quality of life; a-85 respondents to the question regarding assessments but one did not report whether s/he worked in an integrated clinic*.

Overall, 91.0% (71/78) of respondents indicated that they routinely asked about stress, anxiety or depression. This proportion was higher among respondents working within MS Clinics (57/59, 96.6%) than among those who did not (14/19, 73.7%, *p* = 0.0082). When we restricted the analysis to neurologists, this difference was larger (MS Clinic: 100%, non-MS Clinic: 0%, *p* = 0.0008).

### Ideal Models of Care

**Figure 4:**
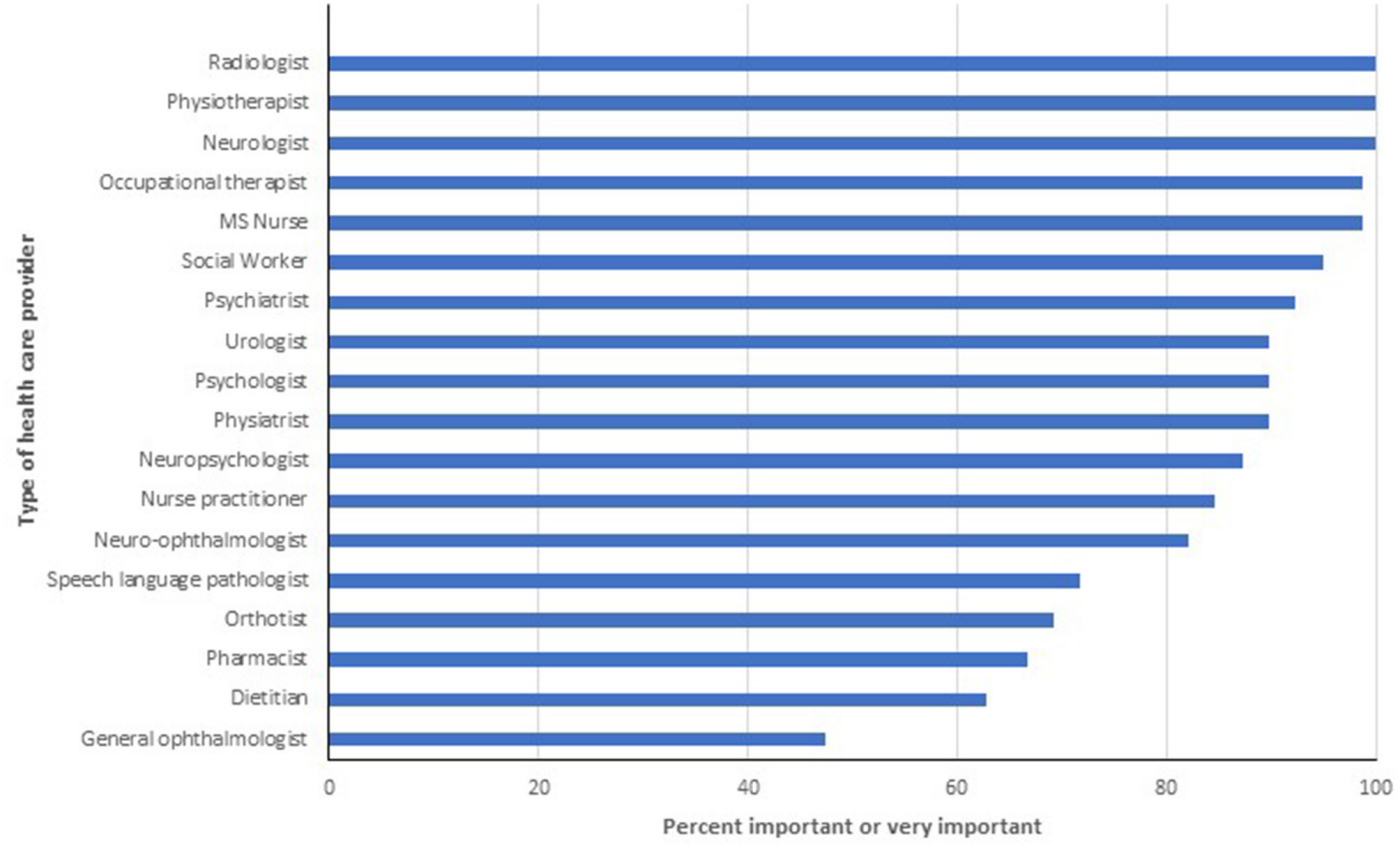
Percentage of respondents indicating provider is important or very important for high quality multiple sclerosis care.

**Figure 5:**
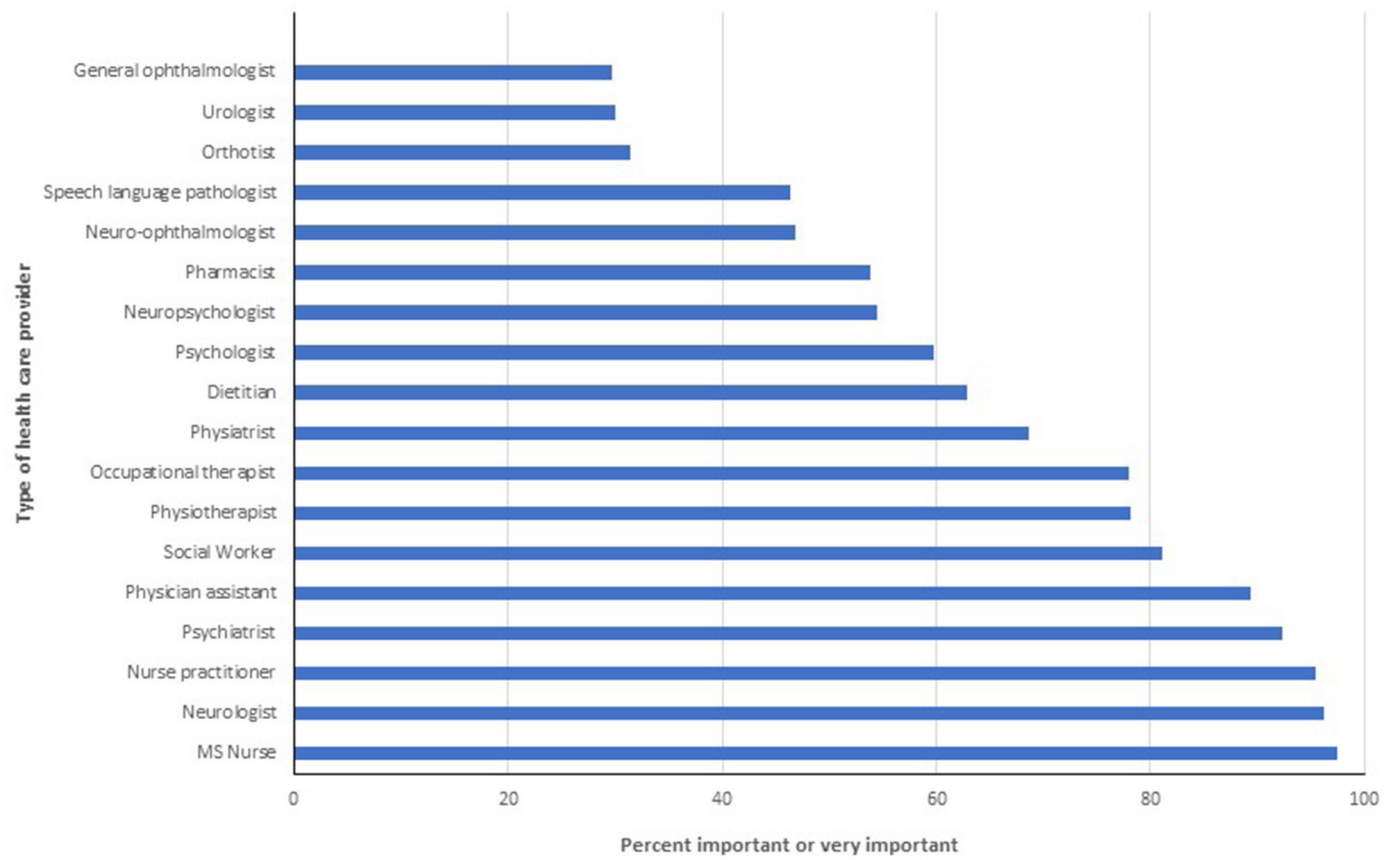
Percentage of respondents indicating it is important or very important that the provider be located within a multiple sclerosis clinic.

Multiple disciplines were identified as important or very important for delivering good quality MS care (Figure 4). Only speech language pathologists (71.8%), orthotists (69.2%) and pharmacists (66.7%) were considered important or very important by fewer than 80% of respondents. Responses to the follow-up question indicated that it was important or very important for most types of HCPs queried to be working within an MS Clinic (Figure 5). Specifically, over 90% of respondents thought it was important for neurologists, nurse practitioners, MS nurses and psychiatrists to work within MS Clinics, and 75–89% thought it was important for occupational therapists, physiotherapists and social workers. In contrast, fewer than one-third of respondents thought that general ophthalmologists, urologists or orthotists needed to work within an MS Clinic. We did not identify any differences in responses across disciplines (all *p* > 0.05).

## Discussion

In this cross-sectional study we surveyed health care professionals, primarily neurologists, caring for people with MS across Canada. Over 40% of MS Clinics reported that they did not have enough neurologists to provide adequate care and nearly 70% of clinics reported that they did not have enough non-neurologist professionals to provide adequate care. More than half of MS Clinics reported wait times longer than 3 months for multiple types of providers including physiatrists, physiotherapists, psychiatrists, psychologists, neuropsychologists and urologists. However, multiple disciplines were perceived as important or very important for delivering good quality care. The ideal MS service was described as multidisciplinary, adequately staffed without time constraints for patient care, and systematic assessments of patient outcomes. Routinely performed assessments most often included the EDSS and screening for symptoms of stress, depression and anxiety. Although 81% of the MS Clinics represented reported practicing in an integrated model of care, and nearly all integrated clinics had neurologists and MS nurses, the remaining complement of HCPs was not consistent across clinics.

Individuals living with MS may suffer from a plethora of symptoms including weakness, sensory symptoms, bowel and bladder dysfunction, fatigue, spasticity, pain, and cognitive impairment. This was reflected in the widespread agreement that health care professionals from multiple disciplines are needed to provide good quality care for people living with MS. However, our survey suggests substantial variability with respect to the types of providers that are readily accessible to people living with MS, whether internal to or external to formal MS Clinics. Nearly all MS Clinics had access to neurologists and MS nurses but timely access to providers, as defined by wait times for referrals of <3 months, was more limited including for neurologists. The 2021 Atlas of MS reported that unmet needs for rehabilitation and symptom management were high (16), but was unable to discriminate between availability of providers vs. ability to access them in a timely fashion. Our findings suggest that in a Canadian context, both availability and timely access are a concern. Further, the 2021 Atlas of MS reported that availability of therapy for impaired mobility and spasticity was greater than for fatigue and cognitive impairment, mirroring the more limited access to occupational therapy and neuropsychology that we observed.

A European colloquium did not reach agreement regarding the structural organization of MS care teams and whether they needed to be co-localized (17). The ECTRIMS-EAN guidelines similarly recommend that the full spectrum of DMTs be provided only in centres (e.g., specialized MS Clinics) with adequate expertise and resources to provide appropriate assessments, monitoring and the ability to address adverse effects (18). Our findings demonstrate that there is no standard model of care across Canada, and also highlight a gap between current practice models and perspectives of ideal care models. Respondents indicated that the ideal MS service would be adequately staffed, and multidisciplinary, involving neurologists, nurses, psychiatrists, social workers, physiotherapists and occupational therapists, to provide timely integrated comprehensive care. Routine assessments at regular visits, and adequate time to spend with patients were also described as key components of an ideal MS service. In 2019, Sorensen et al. promoted the need for comprehensive “MS Care Units” to ensure early diagnosis, provide timely access to the full spectrum of interventions for care, including DMT, support shared decision-making, and provide appropriate monitoring and risk mitigation (9). The core of these MS Care Units was proposed to be MS neurologists and nurses, at least three of neuropsychologists, clinical psychologists, physiotherapists, occupational therapists, speech therapists, social workers as well as specialist services related to diet, management of spasticity, incontinence and pain.

Findings in this survey regarding the ideal MS service, and poor access to the full range of providers expressed by HCPs are concordant with concerns expressed by people living with MS in other studies. A survey of 324 Canadians with MS found that two-thirds reported that their neurologist was their main source of MS care, but had difficulty accessing their neurologist as often as they wished (19). Occupational therapists, mental health providers and physiotherapists were the top HCPs whom participants needed to see but could not access. Encountering providers who lacked knowledge about MS and understood their situations was also a significant concern (19), echoed in a related qualitative study (20) and in a second qualitative study among moderately to severely affected individuals with MS in Germany (21). A recent survey of 1,190 persons, 75% of whom had MS, identified the influence of multidisciplinary teams on health outcomes and experiences as one of the top five research priorities (22). A study involving 707 patients from 81 centers in Italy found that patient satisfaction was lower in larger centres, and higher when a centre provided access to psychotherapy, suggesting a widespread need for mental health supports (23).

Our findings should be interpreted in light of limitations. We did not include a random sample of all clinicians delivering care to persons with MS. Further, the response rate was low despite the use of multiple reminders as recommended (24), potentially causing selection bias. The low response was likely influenced by several factors. First, response rates to electronic surveys have declined over time (25). Second, physicians who constituted the largest proportion of professionals in the sampling frame are known to have low response rates. We intentionally tried to capture neurologists who might deliver MS care outside MS clinics to gather a range of perspectives but found that very few neurologists who did not deliver MS care responded to the survey. This is consistent with prior observations that potential respondents are more likely to complete a survey when it is of high interest to them (24). Third, the survey was distributed during a period when a wave of the COVID-19 pandemic was placing substantial demands on Canadian health care professionals, some of whom who were assigned additional or alternative clinical responsibilities, which may have further reduced response rates. Third, we learned that some institutional spam filters were classifying the invitations as junk or blocking them altogether; it is not known how many invitations were adversely affected by this issue. We sought to mitigate this issue by subsequently issuing each email reminder manually (26). However, the response rate for MS Clinics regarding their models of care, including composition of clinics, timelines of care, and database practices was 80.8%. It is unknown if our findings would generalize to other health systems. We investigated the existence of multidisciplinary models of care, but did not assess the existence of integrated care pathways, which are designed to provide a clear pathway for timely delivery of multidisciplinary care for a specific symptom or condition. Responses regarding wait times were informed by available wait time data in some but not all MS clinics, which may have affected accuracy of those responses. Comparisons between integrated and non-integrated clinics should be viewed cautiously, given the small number of non-integrated clinics/services that responded. We captured the perspectives of HCPs regarding the ideal MS service which may be influenced by the types of providers and models of care to which they have been exposed. Our list of potential providers within MS services did not include all possible providers, such as those offering palliative care. In 2020, the European Academy of Neurology proposed that home-based palliative care be offered to individuals living with severe progressive MS, although the quality of evidence supporting this statement was weak (27). In the United States inpatient palliative care remains uncommon, with only 6.1% of hospitalized people with MS receiving it in 2014 (28). A relatively low proportion of Canadians (<15%) receive palliative care in their last year of life, even among those receiving long-term care (22%) (29). Future studies should evaluate the role and integration of palliative care providers in MS Clinics. We also did not address the role of primary care providers because they are not usually integrated within MS Clinics, but they are key members of the larger care team. Finally, we did not capture the perspectives of people living with MS or their caregivers who report unmet needs (30), but prior studies in the Canadian setting are available.

## Conclusion

Canadian HCPs viewed the ideal MS service as being multidisciplinary in nature, ideally integrated, with timely access to care. This is concordant with needs identified by people living with MS, which highlights the importance and urgency of ensuring availability of these models of care. Substantial variability existed in the types of providers situated within MS Clinics, and in the types of providers who are accessible outside MS Clinics. Wait times for were also highly variable but exceeded 3 months in many centres for multiple types of providers. Efforts are needed to improve access to specialized MS care in Canada, and to evaluate how outcomes are influenced by access to care.

## Data Availability Statement

The datasets presented in this article are not readily available because the survey consent statement did not indicate data would be shared beyond study investigators. Requests to access the datasets should be directed to rmarrie@hsc.mb.ca.

## Ethics Statement

The studies involving human participants were reviewed and approved by University of Manitoba Health Research Ethics Board and Shared Health. Written informed consent for participation was not required for this study in accordance with the national legislation and the institutional requirements.

## Author Contributions

RM: conceptualization, methodology, data curation, formal analysis, writing—original draft, writing—review and editing, and supervision. SD and JP: conceptualization, methodology, and writing—review and editing. DJ, JO, LM, PS, VD, OH, and SM: conceptualization and writing—review and editing. All authors reviewed the draft, provided feedback, and approved the final manuscript.

## Funding

This study was funded in part by the Waugh Family Chair in Multiple Sclerosis. The funding source had no role in the design, implementation or analysis of the survey.

## Conflict of Interest

RM receives research funding from: CIHR, Research Manitoba, Multiple Sclerosis Society of Canada, Multiple Sclerosis Scientific Foundation, Crohn's and Colitis Canada, National Multiple Sclerosis Society, CMSC. She is a co-investigator on a study funded in part by Biogen Idec and Roche Canada. She is supported by the Waugh Family Chair in Multiple Sclerosis. SD receives research funding from the: Canadian Institute of Health Research, Multiple Sclerosis Society of Canada, Saskatchewan Health Research Foundation, Saskatchewan Centre for Patient Oriented Research, Saskatchewan Ministry of Health, Branch Out Neurological Foundation, Praxis Spinal Cord Institute, and Canadian Partnership for Stroke Recovery. LM receives research funding from the MS Society of Canada, Brain Canada, Roche, Biogen Idec and the Government of Alberta. SM has in the past 3 years served on advisory boards for Biogen Idec, Bristol Myers Squibb/Celgene, EMD Serono, Novartis, Roche, Sanofi Genzyme, Teva Neurosciences; Received Investigator Initiated Grant Funds from MS Society of Canada, National MS Society and CIHR, as well as Biogen Idec, Novartis, Roche, Sanofi Genzyme; Acted as site PI for multi-center trials funded by AbbVie, Bristol Myers Squibb/Celgene, Novartis, Genzyme, Roche, Sanofi Genzyme. JO has received research funding from the MS Society of Canada, National MS Society, NIH, Brain Canada, Biogen-Idec, Roche, and EMD-Serono. She has received personal compensation for consulting or speaking from Biogen-Idec, BMS, EMD-Serono, Novartis, Roche, Sanofi-Genzyme, Roche, and Alexion. She is supported by the Waugh Family Chair in MS Research at the University of Toronto. PS has received unrestricted research support from Biogen-Idec; and has received consulting fees and honoraria for speaking activities from Biogen-Idec and Roche. She receives research funding from the MS Society of Canada, Brain Canada, Roche, Biogen Idec and the Government of Alberta. VD has received honoraria for speaking/advisory boards from Biogen, Serono, Roche, Sanofi-Genzyme, Novartis and Teva Neuroscience. The remaining authors declare that the research was conducted in the absence of any commercial or financial relationships that could be construed as a potential conflict of interest.

## Publisher's Note

All claims expressed in this article are solely those of the authors and do not necessarily represent those of their affiliated organizations, or those of the publisher, the editors and the reviewers. Any product that may be evaluated in this article, or claim that may be made by its manufacturer, is not guaranteed or endorsed by the publisher.
