# Supplementary material for: Models of Care in Multiple Sclerosis: A Survey of Canadian Health Providers
Source: Front Neurol. 2022 May 20;13:904757. doi: 10.3389/fneur.2022.904757 (PMC9163821; doi:10.3389/fneur.2022.904757)
Supplement: Supplementary file 1 [file Data_Sheet_1.PDF]

Models of Care in Multiple Sclerosis

RESEARCH PARTICIPANT INFORMATION AND CONSENT FORM

Title of Study: Models of Care in Multiple Sclerosis

Principal Investigator (University of Manitoba):

Dr. Ruth Ann Marrie
GF- 543 Health Sciences Centre,
820 Sherbrook Street,
Winnipeg, MB R3A 1R9

Purpose of Study

The purpose of this research project is to understand the current models of care for multiple sclerosis in Canada, to assess perceptions of health care providers regarding the models of care required to meet the needs of persons with MS. This is a research project being led by Dr. Ruth Ann Marrie at the University of Manitoba. You are invited to participate in this research project because you are a health care provider who cares for people living with MS.

Participation

If you decide to participate you will fill out an online survey that will take approximately 20-25 minutes. Your responses will be confidential and we will not collect identifying information such as your name, or address. Your email address for this online survey will not be linked with your responses. You may save a partially completed survey and return later to complete it. To do so, save select 'Save and Return Later' then write down the code that is displayed. You will be asked to enter it when you return to the survey.

You will not receive any compensation for participation and there are no direct benefits to your participation. There are no foreseeable risks to your participation.

Confidentiality

We will do our best to keep your information confidential. Because we are contacting you via email it is possible that you could be identified. However, we will minimize this risk in two ways - we will not link your email address to your responses, and we will delete all email addresses once data collection is complete. All data will be stored in a password protected electronic REDCap database. The results of this study will be used for scholarly purposes only and may be shared with University of Manitoba representatives.

Questions

If you have any questions about the research study, please contact Dr. Ruth Ann Marrie at 204-787-4778. This research has been reviewed according to University of Manitoba REB procedures for research involving human subjects.

Voluntary

Your participation in this research study is voluntary. You may choose not to participate. If you decide not to participate in this study, you will not be penalized.

By completing this online questionnaire you are consenting to participate.

V2.0 May 2, 2021 Page 1 of 1

[Attachment: "Study information letter and disclosure statement_V2.0.pdf"]

About You

1.What is your gender?

◯ Female
◯ Male
◯ Neither male nor female
◯ Prefer not to answer

2. What is your age in years?

____

3. What is your discipline?

◯ Neurologist ◯ Physiatrist ◯ MS Nurse ◯ Nurse practitioner ◯ Physician assistant ◯ Physiotherapist ◯ Occupational therapist ◯ Social worker ◯ Psychologist ◯ Psychiatrist/Neuropsychiatrist ◯ Neuroradiologist/Radiologist ◯ Dietician ◯ Urologist/Urogynecologist ◯ Ophthalmologist (general) ◯ Neuro-ophthalmologist ◯ Speech-language pathologist ◯ Pharmacist ◯ Neuropsychologist ◯ Other (Specify below)

Please specify:

____

4. Do you have a particular interest in MS?
(If no, skip to question 6)

◯ Yes
◯ No

5. Do you have fellowship training in MS?

◯ Yes
◯ No

6. Does your clinical practice include people living with MS?

◯ Yes
◯ No

7. What percentage of your clinical work concerns MS?

0 50 100

(Place a mark on the scale above)

8. How many years following completion of your training have you been involved in MS health care?

◯ 0
◯ 1
◯ 2
◯ 3
◯ 4
◯ 5
◯ 6
◯ 7
◯ 8
◯ 9
◯ 10
◯ 11
◯ 12
◯ 13
◯ 14
◯ 15
◯ 16
◯ 17
◯ 18
◯ 19
◯ 20
◯ 21
◯ 22
◯ 23
◯ 24
◯ 25
◯ 26
◯ 27
◯ 28
◯ 29
◯ 30
◯ 31
◯ 32
◯ 33
◯ 34
◯ 35
◯ 36
◯ 37
◯ 38
◯ 39
◯ 40
◯ 41
◯ 42
◯ 43
◯ 44
◯ 45
◯ 46
◯ 47
◯ 48
◯ 49
◯ 50

9. How many MS patients do you have contact with (face to face or virtual) on average each week?

____

10. How many MS patients do you provide ongoing care to?

____

Your work environment

Progress

11. What province or territory do you primarily work in?

◯ British Columbia ◯ Alberta ◯ Saskatchewan ◯ Manitoba ◯ Ontario ◯ Quebec
◯ New Brunswick ◯ Nova Scotia ◯ Prince Edward Island ◯ Newfoundland ◯ Yukon
◯ Northwest Territories ◯ Nunavut

12. What is the setting of your MS practice? (Choose all that apply)

☐ General hospital
☐ University hospital
☐ Solo private practice
☐ Group private practice
☐ Other (specify)

Other (please specify)

____

13. Do you treat adults with MS?

◯ Yes
◯ No

14. Do you treat children (aged 16 years or under) with MS?

◯ Yes
◯ No

15. Is your MS service located within a formally labelled MS Clinic?

◯ Yes
◯ No

16. Please provide the name of your clinic:

◯ Alberta: Multiple Sclerosis Clinic Foothills Hospital ◯ Alberta: Multiple Sclerosis Patient Care and Research Clinic
◯ Alberta: Red Deer MS clinic - Red Deer Regional Hospital Centre
◯ BC: UBC Hospital ◯ BC: Vancouver Island MS Clinic Royal Jubilee Hospital
◯ BC: Fraser Health Multiple Sclerosis Clinic Burnaby Hospital ◯ BC: Satellite Clinic of UBC (Associate) Kelowna General Hospital
◯ BC: Satellite Clinic of UBC MS Clinic Prince George Regional Hospital
◯ Manitoba: Health Sciences Centre
◯ New Brunswick: Horizon Health Network Multiple Sclerosis Clinic Saint John Regional Hospital, 5DN ◯ New Brunswick: The Moncton Hospital, Clinic A-Ambulatory Care
◯ Newfoundland: MS Clinic, Division of Neurology ◯ Nova Scotia: Dalhousie Multiple Sclerosis Research Unit
◯ Nova Scotia: Neurology Clinic Coordinator Cape Breton Regional Hospital
◯ Ontario: St. Michael's Hospital MS Clinic, MS Research Centre ◯ Ontario: Sunnybrook Health Sciences Centre (Associate)
◯ Ontario: The Hospital for Sick Children Pediatric Demyelinating Disease Program Division of Neurology ◯ Ontario, Kingston MS Clinic Kingston General Hospital
◯ Ontario, Hamilton Health Sciences Hamilton General Hospital ◯ Ontario, MS Clinic - Guelph ◯ Ontario, London Health Sciences Centre - University Hospital London MS Clinic
◯ Ontario, MS Research Clinic Ottawa Hospital General Campus ◯ Ontario: Pediatric MS Clinic Clinic C9 Children's Hospital of Eastern Ontario ◯ Ontario: Thunder Bay MS Clinic
◯ Quebec: CHUM Hôpital Notre-Dame
◯ Quebec: Montreal Neurological Institute
◯ Quebec: Clinique de sclérose en plaques et des maladies neuromusculaires CIUSSS Capitale-Nationale, IRDPQ
◯ Quebec: Neuro Rive-Sud MS Clinic
◯ Quebec: Vallées de l'Outaouais Hospital
◯ Quebec: Clinique de SP, Mauricie-Bois-Francs Cœur du Québec
◯ Quebec: Clinique de sclérose en plaques du CIUSSS de l'Estrie - CHUS
◯ Quebec: Clinique de sclérose en plaques, CRDP Chaudière-Appalaches - Levis
◯ Saskatchewan: Saskatoon City Hospital
◯ Other (Please specify)

Other: Please specify clinic name

____

17. You indicated that you work in an MS Clinic. In advance of this survey, we asked each clinic to designate one person to answer questions #18-20. Are you that person?
(Yes - go to #18 No - go to #21)

◯ Yes
◯ No

18. Do you consider your MS service to apply an integrated model of care (i.e. a model in which several health care providers are located at the same site and manage patients collaboratively)?
(If yes, go to question #19, if no go to #20)

◯ Yes
◯ No

Progress

19. What types of health professionals provide MS care within your MS service?

	No	Yes
19 a. Neurologist	◯	◯
19 b. MS Nurse	◯	◯
19 c. Nurse practitioner	◯	◯
19 d. Physician assistant	◯	◯
19 e. Physiotherapist	◯	◯
19 f. Occupational therapist	◯	◯
19 g. Social worker	◯	◯
19 h. Psychologist	◯	◯
19 i. Psychiatrist/Neuropsychiatrist	◯	◯
19 j. Radiologist/Neuroradiologist	◯	◯
19 k. Dietitian	◯	◯
19 l. Urologist/Urogynecologist	◯	◯
19 m. Ophthalmologist (general)	◯	◯
19 n. Neuro-opthalmologist	◯	◯
19 o. Speech-language pathologist	◯	◯
19 p. Physiatrist	◯	◯
19 q. Pharmacist	◯	◯
19 r. Neuropsychologist	◯	◯
19 s. Orthotist	◯	◯

19a. Neurologists:
i) What is the wait time for a new referral?

◯ Not applicable ◯ 0-3 months ◯ 4-6 months ◯ 7-12 months ◯ > 12 months

19a. Neurologists:
ii) What is the number of neurologist professionals?

◯ 1 ◯ 2 ◯ 3 ◯ 4
◯ 5 ◯ 6 ◯ 7 ◯ 8
◯ 9 ◯ 10 ◯ 11 or more

19a. Neurologists:
iii) What is the total full-time equivalents (FTE)?

____

19b. MS Nurse:
i) What is the wait time for a new referral?

◯ Not applicable ◯ 0-3 months ◯ 4-6 months ◯ 7-12 months ◯ > 12 months

---

19b. MS Nurses:
ii) What is the number of MS Nurse professionals?

◯ 1 ◯ 2 ◯ 3 ◯ 4
◯ 5 ◯ 6 ◯ 7 ◯ 8
◯ 9 ◯ 10 ◯ 11 or more

19b. MS Nurses:
iii) What is the total full-time equivalents (FTE)?

____

19c. Nurse Practitioners:
i) What is the wait time for a new referral?

◯ Not applicable ◯ 0-3 months ◯ 4-6 months ◯ 7-12 months ◯ > 12 months

---

19c. Nurse practitioners:
ii) What is the number of Nurse practitioner professionals?

◯ 1 ◯ 2 ◯ 3 ◯ 4
◯ 5 ◯ 6 ◯ 7 ◯ 8
◯ 9 ◯ 10 ◯ 11 or more

19c. Nurse practitioners:
iii) What is the total full-time equivalents (FTE)?

____

19d. Physician Assistant:
i) What is the wait time for a new referral?

◯ Not applicable ◯ 0-3 months ◯ 4-6 months ◯ 7-12 months ◯ > 12 months

---

19d. Physician assistants:
ii) What is the number of physician assistant professionals?

◯ 1 ◯ 2 ◯ 3 ◯ 4
◯ 5 ◯ 6 ◯ 7 ◯ 8
◯ 9 ◯ 10 ◯ 11 or more

19d. Physician assistants:
iii) What is the total full-time equivalents (FTE)?

____

19e. Physiotherapist:
i) What is the wait time for a new referral?

◯ Not applicable ◯ 0-3 months ◯ 4-6 months ◯ 7-12 months ◯ > 12 months

---

19e. Physiotherapist:
ii) What is the number of physiotherapist professionals?

◯ 1 ◯ 2 ◯ 3 ◯ 4
◯ 5 ◯ 6 ◯ 7 ◯ 8
◯ 9 ◯ 10 ◯ 11 or more

19e. Physiotherapists:
iii) What is the total full-time equivalents (FTE)?

____

19f. Occupational Therapists:
i) What is the wait time for a new referral?

◯ Not applicable ◯ 0-3 months ◯ 4-6 months ◯ 7-12 months ◯ > 12 months

19f. Occupational Therapists:
ii) What is the number of Occupational Therapists professionals?

◯ 1 ◯ 2 ◯ 3 ◯ 4
◯ 5 ◯ 6 ◯ 7 ◯ 8
◯ 9 ◯ 10 ◯ 11 or more

19f. Occupational Therapists:
iii) What is the total full-time equivalents (FTE)?

____

19g. Social Workers:
i) What is the wait time for a new referral?

◯ Not applicable ◯ 0-3 months ◯ 4-6 months ◯ 7-12 months ◯ > 12 months

19g. Social Workers:
ii) What is the number of Social Worker professionals?

◯ 1 ◯ 2 ◯ 3 ◯ 4
◯ 5 ◯ 6 ◯ 7 ◯ 8
◯ 9 ◯ 10 ◯ 11 or more

19g. Social Workers:
iii) What is the total full-time equivalents (FTE)?

____

19h. Psychologist:
i) What is the wait time for a new referral?

◯ Not applicable ◯ 0-3 months ◯ 4-6 months ◯ 7-12 months ◯ > 12 months

19h. Psychologists:
ii) What is the number of Psychologist professionals?

◯ 1 ◯ 2 ◯ 3 ◯ 4
◯ 5 ◯ 6 ◯ 7 ◯ 8
◯ 9 ◯ 10 ◯ 11 or more

19h. Psychologists:
iii) What is the total full-time equivalents (FTE)?

____

19i. Psychiatrists/Neuropsychiatrists:
i) What is the wait time for a new referral?

◯ Not applicable ◯ 0-3 months ◯ 4-6 months ◯ 7-12 months ◯ > 12 months

19i. Psychiatrists/Neuropsychiatrist:
ii) What is the number of Psychiatrist/Neuropsychiatrist professionals?

◯ 1 ◯ 2 ◯ 3 ◯ 4
◯ 5 ◯ 6 ◯ 7 ◯ 8
◯ 9 ◯ 10 ◯ 11 or more

19i. Psychiatrist/Neuropsychiatrist:
iii) What is the total full-time equivalents (FTE)?

____

19j. Radiologists/Neuroradiologists:
i) What is the wait time for a new referral?

○ Not applicable ○ 0-3 months ○ 4-6 months ○ 7-12 months ○ > 12 months

19j. Radiologists/Neuroradiologist:
ii) What is the number of Radiologist/Neuroradiologist professionals?

○ 1 ○ 2 ○ 3 ○ 4
○ 5 ○ 6 ○ 7 ○ 8
○ 9 ○ 10 ○ 11 or more

19j: Radiologists/Neuroradiologists:
iii) What is the total full-time equivalents (FTE)?

____

19k. Dietitians:
i) What is the wait time for a new referral?

○ Not applicable ○ 0-3 months ○ 4-6 months ○ 7-12 months ○ > 12 months

19k. Dieticians:
ii) What is the number of Dietician professionals?

○ 1 ○ 2 ○ 3 ○ 4
○ 5 ○ 6 ○ 7 ○ 8
○ 9 ○ 10 ○ 11 or more

19k. Dieticians:
iii) What is the total full-time equivalents (FTE)?

____

19l. Urologists/Urogynecologists:
i) What is the wait time for a new referral?

○ Not applicable ○ 0-3 months ○ 4-6 months ○ 7-12 months ○ > 12 months

19l. Urologists/Urogynecologists:
ii) What is the number of Urologist/Urogynecologists professionals?

○ 1 ○ 2 ○ 3 ○ 4
○ 5 ○ 6 ○ 7 ○ 8
○ 9 ○ 10 ○ 11 or more

19l. Urologists/Urogynecologists:
iii) What is the total full-time equivalents (FTE)?

____

19m. Ophthalmologists (general):
i) What is the wait time for a new referral?

○ Not applicable ○ 0-3 months ○ 4-6 months ○ 7-12 months ○ > 12 months

19m. Ophthalmologists (general):
ii) What is the number of Ophthalmologist professionals?

○ 1 ○ 2 ○ 3 ○ 4
○ 5 ○ 6 ○ 7 ○ 8
○ 9 ○ 10 ○ 11 or more

19m. Ophthalmologists (general):
iii) What is the total full-time equivalents (FTE)?

____

19n. Neuro-ophthalmologists:
i) What is the wait time for a new referral?

◯ Not applicable ◯ 0-3 months ◯ 4-6 months ◯ 7-12 months ◯ > 12 months

19n. Neuro-ophthalmologists:
ii) What is the number of Neuro-ophthalmologist professionals?

◯ 1 ◯ 2 ◯ 3 ◯ 4
◯ 5 ◯ 6 ◯ 7 ◯ 8
◯ 9 ◯ 10 ◯ 11 or more

19n. Neuro-ophthalmologists:
iii) What is the total full-time equivalents (FTE)?

____

19o. Speech-language pathologists:
i) What is the wait time for a new referral?

◯ Not applicable ◯ 0-3 months ◯ 4-6 months ◯ 7-12 months ◯ > 12 months

19o. Speech-language pathologists:
ii) What is the number of Speech-language pathologist professionals?

◯ 1 ◯ 2 ◯ 3 ◯ 4
◯ 5 ◯ 6 ◯ 7 ◯ 8
◯ 9 ◯ 10 ◯ 11 or more

19o. Speech-language pathologists:
iii) What is the total full-time equivalents (FTE)?

____

19p. Physiatrists:
i) is the wait time for a new referral?

◯ Not applicable ◯ 0-3 months ◯ 4-6 months ◯ 7-12 months ◯ > 12 months

19p. Physiatrists:
ii) What is the number of Physiatrist professionals?

◯ 1 ◯ 2 ◯ 3 ◯ 4
◯ 5 ◯ 6 ◯ 7 ◯ 8
◯ 9 ◯ 10 ◯ 11 or more

19p. Physiatrists:
iii) What is the total full-time equivalents (FTE)?

____

19q. Pharmacists:
i) What is the wait time for a new referral?

◯ Not applicable ◯ 0-3 months ◯ 4-6 months ◯ 7-12 months ◯ > 12 months

19q. Pharmacists:
ii) What is the number of Pharmacist professionals?

◯ 1 ◯ 2 ◯ 3 ◯ 4
◯ 5 ◯ 6 ◯ 7 ◯ 8
◯ 9 ◯ 10 ◯ 11 or more

19p. Pharmacists:
iii) What is the total full-time equivalents (FTE)?

____

19r. Neuropsychologists:
i) What is the wait time for a new referral?

◯ Not applicable ◯ 0-3 months ◯ 4-6 months ◯ 7-12 months ◯ > 12 months

---

19r. Neuropsychologists:
ii) What is the number of Neuropsychologist professionals?

◯ 1 ◯ 2 ◯ 3 ◯ 4
◯ 5 ◯ 6 ◯ 7 ◯ 8
◯ 9 ◯ 10 ◯ 11 or more

19r. Neuropsychologists:
iii) What is the total full-time equivalents (FTE)?

____

19s. Orthotists:
i) What is the wait time for a new referral?

◯ Not applicable ◯ 0-3 months ◯ 4-6 months ◯ 7-12 months ◯ > 12 months

---

19s. Orthotists:
ii) What is the number of Orthotist professionals?

◯ 1 ◯ 2 ◯ 3 ◯ 4
◯ 5 ◯ 6 ◯ 7 ◯ 8
◯ 9 ◯ 10 ◯ 11 or more

19s. Orthotists:
iii) What is the total full-time equivalents (FTE)?

____

Progress

20. What types of health professionals outside your MS service are accessible to your patients?

	No	Yes
20 a. Neurologist	◯	◯
20 b. MS Nurse	◯	◯
20 c. Nurse Practitioner	◯	◯
20 d. Physician assistant	◯	◯
20 e. Physiotherapist	◯	◯
20 f. Occupational Therapist	◯	◯
20 g. Social Worker	◯	◯
20 h. Psychologist	◯	◯
20 i. Psychiatrist/Neuropsychiatrist	◯	◯
20 j. Radiologist/neuroradiologist	◯	◯
20 k. Dietician	◯	◯
20 l. Urologist/Urogynecologist	◯	◯
20 m. General Ophthalmologist	◯	◯
20 n. Neuro-ophthalmologist	◯	◯
20 o. Speech language pathologist	◯	◯

20 p. Physiatrist	○	○
20 q. Pharmacist	○	○
20 r. Neuropsychologist	○	○
20 s. Orthotist	○	○

20a. Neurologist: What is the wait time for a new referral?

○ Not applicable ○ 0-3 months ○ 4-6 months ○ 7-12 months ○ > 12 months

---

20b. MS Nurse: What is the wait time for a new referral?

○ Not applicable ○ 0-3 months ○ 4-6 months ○ 7-12 months ○ > 12 months

---

20c. Nurse Practitioner: What is the wait time for a new referral?

○ Not applicable ○ 0-3 months ○ 4-6 months ○ 7-12 months ○ > 12 months

---

20d. Physician assistant: What is the wait time for a new referral?

○ Not applicable ○ 0-3 months ○ 4-6 months ○ 7-12 months ○ > 12 months

---

20e. Physiotherapist: What is the wait time for a new referral?

○ Not applicable ○ 0-3 months ○ 4-6 months ○ 7-12 months ○ > 12 months

---

20f. Occupational Therapist: What is the wait time for a new referral?

○ Not applicable ○ 0-3 months ○ 4-6 months ○ 7-12 months ○ > 12 months

---

20g. Social Worker : What is the wait time for a new referral?

○ Not applicable ○ 0-3 months ○ 4-6 months ○ 7-12 months ○ > 12 months

---

20h. Psychologist : What is the wait time for a new referral?

○ Not applicable ○ 0-3 months ○ 4-6 months ○ 7-12 months ○ > 12 months

---

20i. Psychiatrist/Neuropsychiatrist: What is the wait time for a new referral?

○ Not applicable ○ 0-3 months ○ 4-6 months ○ 7-12 months ○ > 12 months

---

20j. Radiologist/Neuroradiologist: What is the wait time for a new referral?

○ Not applicable ○ 0-3 months ○ 4-6 months ○ 7-12 months ○ > 12 months

---

20k. Dietician: What is the wait time for a new referral?

○ Not applicable ○ 0-3 months ○ 4-6 months ○ 7-12 months ○ > 12 months

20l. Urologist/Urogynecologist: What is the wait time for a new referral?

◯ Not applicable ◯ 0-3 months ◯ 4-6 months ◯ 7-12 months ◯ > 12 months

20m. General ophthalmologist: What is the wait time for a new referral?

◯ Not applicable ◯ 0-3 months ◯ 4-6 months ◯ 7-12 months ◯ > 12 months

20n. Neuro-ophthalmologist: What is the wait time for a new referral?

◯ Not applicable ◯ 0-3 months ◯ 4-6 months ◯ 7-12 months ◯ > 12 months

20o. Speech-language pathologist: What is the wait time for a new referral?

◯ Not applicable ◯ 0-3 months ◯ 4-6 months ◯ 7-12 months ◯ > 12 months

20p. Physiatrist: What is the wait time for a new referral?

◯ Not applicable ◯ 0-3 months ◯ 4-6 months ◯ 7-12 months ◯ > 12 months

20q. Pharmacist: What is the wait time for a new referral?

◯ Not applicable ◯ 0-3 months ◯ 4-6 months ◯ 7-12 months ◯ > 12 months

20r. Neuropsychologist: What is the wait time for a new referral?

◯ Not applicable ◯ 0-3 months ◯ 4-6 months ◯ 7-12 months ◯ > 12 months

20s. Orthotist: What is the wait time for a new referral?

◯ Not applicable ◯ 0-3 months ◯ 4-6 months ◯ 7-12 months ◯ > 12 months

20t. From your perspective, do the number of staffed neurologists, FTEs assigned to each neurologist at your MS Clinic, allow for the provision of optimal MS care?

◯ Yes
◯ No
◯ I'd rather not say

20u. From your perspective, do the number of staffed non-neurologist health professionals, FTEs assigned to each health professional, allow for the provision of optimal MS care?

◯ Yes
◯ No
◯ I'd rather not say

Progress

21. How important are these types of health professionals for good quality MS care?

	Not at all important	Unimportant	Neither important nor unimportant	Important	Very important

21 a. Neurologist	○	○	○	○	○
21 b. MS Nurse	○	○	○	○	○
21 c. Nurse practitioner	○	○	○	○	○
21 d. Physician assistant	○	○	○	○	○
21 e. Physiotherapist	○	○	○	○	○
21 f. Occupational therapist	○	○	○	○	○
21 g. Social Worker	○	○	○	○	○
21 h. Psychologist	○	○	○	○	○
21 i. Psychiatrist	○	○	○	○	○
21 j. Radiologist/Neuroradiologist	○	○	○	○	○
21 k. Dietitican	○	○	○	○	○
21 l. Urologist/Urogynecologist	○	○	○	○	○
21 m. General ophthalmologist	○	○	○	○	○
21 n. Neuro-ophthalmologist	○	○	○	○	○
21 o. Speech-language pathologist	○	○	○	○	○
21 p. Physiatrist	○	○	○	○	○
21 q. Pharmacist	○	○	○	○	○
21 r. Neuropsychologist	○	○	○	○	○
21 s. Orthotist	○	○	○	○	○

22. For those healthcare provider(s) in the above question that you selected as "important/very important", how important is it to good quality MS care that they *work within the MS Clinics* ?

	Not at all important	Unimportant	Neither important nor unimportant	Important	Very important
22 t. Neurologist	○	○	○	○	○
22 u. MS Nurse	○	○	○	○	○
22 v. Nurse practitioner	○	○	○	○	○
22 w. Physician assistant	○	○	○	○	○
22 x. Physiotherapist	○	○	○	○	○
22 y. Occupational therapist	○	○	○	○	○
22 z. Social worker	○	○	○	○	○
22 aa. Psychologist	○	○	○	○	○
22 bb. Psychiatrist	○	○	○	○	○
22 cc. Radiologist/Neuroradiologist	○	○	○	○	○
22 dd. Dietician	○	○	○	○	○
22 ee. Urologist/urogynecologist	○	○	○	○	○
22 ff. General ophthalmologist	○	○	○	○	○

22 gg. Neuro-ophthalmologist	○	○	○	○	○
22 hh. Speech-language pathologist	○	○	○	○	○
22 ii. Physiatrist	○	○	○	○	○
22 jj. Pharmacist	○	○	○	○	○
22 kk. Neuropsychologist	○	○	○	○	○
22 ll. Orthotist	○	○	○	○	○

Progress

23. From your perspective, which of the following are included as part of your role as a neurologist versus that of a family physician in providing care to a patient with MS?

	Family Physicians Only	Neurologists Only	Both	Neither
23 a. Referral to Neurologist/MS clinic	○	○	○	○
23 b. Referral to rehabilitation	○	○	○	○
23 c. Referrals to mental health services	○	○	○	○
23 d. Referrals to specialist for MS symptom management	○	○	○	○
23 e. Ordering MRI if MS suspected	○	○	○	○
23 f. MRI monitoring	○	○	○	○
23 g. MS treatment plan selection and management (Disease modifying therapies)	○	○	○	○
23 h. Disease modifying therapy safety monitoring	○	○	○	○
23 i. Disease modifying therapy effectiveness monitoring	○	○	○	○
23 j. Managing MS symptoms	○	○	○	○
23 k. Comorbidity management (mood disorders, hypertension, hyperlipidemia)	○	○	○	○
23 l. Addressing non-MS related health concerns	○	○	○	○
23 m. Health maintenance and promotion (smoking cessation, regular exercise, healthy diet)	○	○	○	○
23 n. Patient education (self-management skills)	○	○	○	○

23 o. Coordinating care ◯ ◯ ◯ ◯

24. Approximately, what percentage of your MS patients do you refer to the following health professionals?

24 a. Physiotherapist 0 50 100

(Place a mark on the scale above)

24 b. Occupational Therapist 0 50 100

(Place a mark on the scale above)

24 c. Social Worker 0 50 100

(Place a mark on the scale above)

24 d. Psychologist 0 50 100

(Place a mark on the scale above)

24 e. Psychiatrist 0 50 100

(Place a mark on the scale above)

24 f. Neuropsychologist 0 50 100

(Place a mark on the scale above)

24 g. Dietician 0 50 100

(Place a mark on the scale above)

25. Does your MS service hold multidisciplinary team meetings?

◯ Yes
◯ No
◯ Not applicable

26. Does your MS service have an electronic MS Database?

◯ Yes
◯ No

27. Does your MS service do research? (Select all that apply)

☐ Yes, initiated by our team members
☐ Yes, we participate in research developed and led by others
☐ No
☐ Other (please specify)

27. Other (please specify)

____

28. Do you routinely ask questions about stress, anxiety, or depression in an MS patient visit?

◯ Yes
◯ No

29. If yes, please specify:

◯ Verbally ask questions
◯ Use questionnaire ◯ Other

29. Other:

____

Progress

30. Quality improvement requires the ability to measure processes and outcomes. Does your clinic currently collect the following data electronically (clinic database or administrative data) to allow determination of outcomes?

Please comment on the completeness and accuracy of the types of data collected.

30 a. Date of symptom onset

☐ Clinic Level Data
☐ For Each Physician
☐ N/A

30 a. Date of symptom onset: Completeness

0% 50% 100%

(Place a mark on the scale above)

30 a. Date of symptom onset: Accuracy

Low Medium High

(Place a mark on the scale above)

30 b. Date of first neurologist encounter

☐ Clinic Level Data
☐ For Each Physician
☐ N/A

30 b. Date of first neurologist encounter: Completeness

0% 50% 100%

(Place a mark on the scale above)

30 b. Date first neurologist encounter: Accuracy

Low Medium High

(Place a mark on the scale above)

30 c. Date of each MRI after symptom onset

☐ Clinic Level Data
☐ For Each Physician
☐ N/A

30 c. Date of each MRI after symptom onset: Completeness

0% 50% 100%

(Place a mark on the scale above)

30 c. Date of each MRI after symptom onset: Accuracy

Low Medium High

(Place a mark on the scale above)

30 d. Date of first MS clinic visit	☐ Clinic Level Data<br>☐ For Each Physician<br>☐ N/A
30 d. Date of first MS clinic visit: Completeness	0% 50% 100%<br>(Place a mark on the scale above)
30 d. Date of first MS clinic visit: Accuracy	Low Medium High<br>(Place a mark on the scale above)
30 e. Date of first DMT discussion	☐ Clinic Level Data<br>☐ For Each Physician<br>☐ N/A
30 e. Date of first DMT discussion: Completeness	0% 50% 100%<br>(Place a mark on the scale above)
30 e. Date of first DMT Discussion: Accuracy	Low Medium High<br>(Place a mark on the scale above)
30 f. Date of first DMT initiation	☐ Clinic Level Data<br>☐ For Each Physician<br>☐ N/A
30 f. Date of first DMT initiation: Completeness	0% 50% 100%<br>(Place a mark on the scale above)
30 f. Date of first DMT initiation: Accuracy	Low Medium High<br>(Place a mark on the scale above)
30 g. Date of Diagnosis	☐ Clinic Level Data<br>☐ For Each Physician<br>☐ N/A
30 g. Date of diagnosis: Completeness	0% 50% 100%<br>(Place a mark on the scale above)
30 g. Date of diagnosis: Accuracy	Low Medium High<br>(Place a mark on the scale above)
30 h. Date DMT insurance effective	☐ Clinic Level Data<br>☐ For Each Physician<br>☐ N/A
30 h. Date DMT insurance effective: Completeness	0% 50% 100%<br>(Place a mark on the scale above)
30 h. Date DMT insurance effective: Accuracy	Low Medium High<br>(Place a mark on the scale above)

30 i. Dates of each visit	☐ Clinic Level Data<br>☐ For Each Physician<br>☐ N/A
30 i. Dates of each visit: Completeness	0% 50% 100%<br>(Place a mark on the scale above)
30 i. Dates of each visit: Accuracy	Low Medium High<br>(Place a mark on the scale above)
30 j. Date of each EDSS	☐ Clinic Level Data<br>☐ For Each Physician<br>☐ N/A
30 j. Dates of each EDSS: Completeness	0% 50% 100%<br>(Place a mark on the scale above)
30 j. Dates of each EDSS: Accuracy	Low Medium High<br>(Place a mark on the scale above)
30 k. Dates of each care provider encounter (and who provided care)	☐ Clinic Level Data<br>☐ For Each Physician<br>☐ N/A
30 k. Dates of each care provider encounter (and who provided care): Completeness	0% 50% 100%<br>(Place a mark on the scale above)
30 k. Dates of each care provider encounter (and who provided care): Accuracy	Low Medium High<br>(Place a mark on the scale above)
30 l. Dates of each DMT started and stopped	☐ Clinic Level Data<br>☐ For Each Physician<br>☐ N/A
30 l. Dates of each DMT started and stopped: Completeness	0% 50% 100%<br>(Place a mark on the scale above)
30 l. Dates of each DMT started and stopped: Accuracy	Low Medium High<br>(Place a mark on the scale above)
30 m. Reason for DMT switch	☐ Clinic Level Data<br>☐ For Each<br>☐ N/A
30 m. Reason for DMT switch: Completeness	0% 50% 100%<br>(Place a mark on the scale above)

30 m. Reason for DMT switch: Accuracy	Low Medium High<br>(Place a mark on the scale above)
30 n. Dates and scores of each cognitive test (and which test)	☐ Clinic Level Data<br>☐ For Each Physician<br>☐ N/A
30 n. Dates and scores of each cognitive test (and which test): Completeness	0% 50% 100%<br>(Place a mark on the scale above)
30 n. Dates and scores of each cognitive test (and which test): Accuracy	Low Medium High<br>(Place a mark on the scale above)
30 o. Dates of each relapse	☐ Clinic Level Data<br>☐ For Each Physician<br>☐ N/A
30 o. Dates of each relapse: Completeness	0% 50% 100%<br>(Place a mark on the scale above)
30 o. Dates of each relapse: Accuracy	Low Medium High<br>(Place a mark on the scale above)
30 p. Referral to MS Clinic date	☐ Clinic Level Data<br>☐ For Each Physician<br>☐ N/A
30 p. Referral to MS Clinic date: Accuracy	Low Medium High<br>(Place a mark on the scale above)
30 p. Referral to MS Clinic date: Completeness	Low Medium High<br>(Place a mark on the scale above)
30 q. Reason for referral	☐ Clinic Level Data<br>☐ For Each Physician<br>☐ N/A
30 q. Reason for referral: Accuracy	Low Medium High<br>(Place a mark on the scale above)
30 q. Reason for referral: Completeness	Low Medium High<br>(Place a mark on the scale above)
30 r. Referral to MS Clinic was internal to institution or external (from community)	☐ Clinic Level Data<br>☐ For Each Physician<br>☐ N/A

30 r. Referral to MS Clinic was internal to institution or external (from community): Accuracy

Low Medium High

(Place a mark on the scale above)

30 r. Referral to MS Clinic was internal to institution or external (from community): Completeness

Low Medium High

(Place a mark on the scale above)

30 s. Health professional who referred

- ☐ Clinic Level Data
- ☐ For Each Physician
- ☐ N/A

30 s. Health professional who referred: Accuracy

Low Medium High

(Place a mark on the scale above)

30 s. Health professional who referred: Completeness

Low Medium High

(Place a mark on the scale above)

31. Which assessments and screening tools do you use routinely in your appointments with your patients? Select all that apply.

- ☐ Nine hole peg test
- ☐ Timed 25 foot walk
- ☐ SDMT
- ☐ Processing speed test
- ☐ EDSS
- ☐ Quality of life questionnaire (specify)
- ☐ PHQ-9
- ☐ HADS
- ☐ CESD
- ☐ Beck Depression Inventory
- ☐ GAD
- ☐ OASIS
- ☐ PROMIS Depression
- ☐ PROMIS Anxiety
- ☐ Other (specify)

31. Quality of life questionnaire - Please specify:

____

31. Other tools:

____

32. Describe the ideal MS service:

33. What resources would be most helpful in improving MS Care at your clinic (e.g. hours for health care providers, treatment guidelines, equipment)

Progress
