# Supplementary material for: Models of Care in Multiple Sclerosis: A Survey of Canadian Health Providers
Source: Front Neurol. 2022 May 20;13:904757. doi: 10.3389/fneur.2022.904757 (PMC9163821; doi:10.3389/fneur.2022.904757)
Supplement: Supplementary file 2 [file Data_Sheet_2.pdf]

Supplementary Material

Appendix II. Data captured by MS Clinics including completeness and accuracy

Data Element	Captured			Completeness	Accuracy
	N/A	Clinical level data	For each physician		
Date of symptom onset	4 (15.4)	12 (46.2)	9 (34.6)	83.5 (79.0-90.0	83.0 (71.0-91.0)
Date of diagnosis	2 (7.8)	13 (50.0)	9 (34.6)	85.0 (75.0-98.0)	82.0 (75.0-100)
Date of first neurologist encounter	4 (15.4)	11 (42.3)	10 (38.5)	90.5 (76.0, 100)	93.0 (77.0-100)
Date of each MRI after symptom onset	6 (23.1)	10 (38.5)	6 (23.1)	97.0 (75.0-100)	96.0 (73.0-100)
Referral to MS Clinic date	6 (23.1)	11 (42.3)	6 (23.1)	91.0 (79.0-100)	94.0 (70.0-99.0)
Reason for referral	8 (30.8)	8 (30.8)	6 (23.1)	81.0 (74.0-99.0)	81.0 (75.0-98.0)
Was MS Clinic referral internal/ external	9 (34.6)	5 (19.2)	10 (38.5)	73.0 (50.0-100)	72.5 (50.0-100)
Health professional who referred	7 (26.9)	11 (42.3)	7 (26.9)	92.5 (50.0-99.0)	93.0 (50-100)
Date of first MS Clinic visit	2 (7.7)	16 (61.5)	6 (23.1)	100 (96.0-100)	100 (91.0-100)
Dates of each visit	2 (7.8)	15 (57.7)	10 (39.5)	97.0 (85.0-100)	98.0 (96.0-100)
Dates of care provider encounters/who provided care	1 (23.1)	11 (42.3)	8 (30.8)	83.5 (76.0-99.0)	85.6 (75.0-95.0)
Dates of each relapse	4 (15.4)	12 (46.2)	10 (38.5)	72.0 (60.0-77.0)	64.5 (54.5-76.0)
Date of each EDSS	2 (7.7)	12 (46.2)	12 (46.2)	76.6 (72.0-100)	79.0 (75.0-100)
Dates and scores of each cognitive test	11 (42.3)	8 (30.8)	4 (15.4)	100 (94.0-100)	99.0 (94.0-100)
Date of first DMT discussion	8 (30.8)	6 (23.1)	8 (30.8)	80.0 (77.0-98.0)	85.0 (75.0-98.0)
Date DMT insurance effective	12 (46.2)	6 (23.1)	4 (15.4)	77.0 (50.0, 85.0)	79.0 (50.0-81.0)
Date DMT initiation	3 (11.5)	14 (53.8)	8 (30.8)	95.0 (75.0-100)	94.5 (76.0-100)
Dates of each DMT started and stopped	3 (11.5)	15 (57.7)	8 (30.8)	85.0 (70.0-100)	81.0 (71.0-99.0)
Reason for DMT switch	5 (15.4)	12 (46.2)	9 (34.6)	81.5 (64.0-84.5)	80.0 (65.0-90.0)

DMT = disease-modifying therapy, MRI = magnetic resonance imaging
